# Speed‐Variable Treadmill Training Improves Hand Motor Function in Individuals With Parkinson’s Disease

**DOI:** 10.1155/padi/6163541

**Published:** 2026-08-14

**Authors:** Lara Shigo, Brittany E. Smith, Angela L. Ridgel

**Affiliations:** ^1^ Kinesiology, SUNY Cortland, Cortland, New York, USA, cortland.edu; ^2^ Exercise Science, Catawba College, Salisbury, North Carolina, USA, unc.edu; ^3^ Exercise Science and Exercise Physiology, Kent State University, Kent, Ohio, USA, kent.edu

## Abstract

Aerobic exercise programs with proprioceptive sensory feedback elements such as split‐belt treadmill training, perturbation treadmill training, and dynamic cycling improve function in people with Parkinson’s disease (PwPD). However, these tools are expensive and not highly accessible. Speed‐variable (SV) treadmill training utilizes small‐amplitude treadmill belt speed fluctuations every 10–60 s, emulating aforementioned paradigms in a more‐accessible design targeted toward home or gym settings. Fourteen PwPD (67.6 ± 6.5 years, 6 female) completed one 30‐min session of SV training and one 30‐min session of normal (N) non‐speed‐varied treadmill training in a counterbalanced design. Motor function was assessed pre‐ and postexercise with KinesiaONE inertial measurement unit validated for assessment of symptoms including tremor, bradykinesia, rhythm, and amplitude of movement. Heart rate (HR) did not differ significantly (*p* = 0.146, *t* = −1.102) across conditions, with average HR in 60%–65% age‐predicted HRmax range. Rating of perceived exertion (RPE) was significantly (*p* = 0.020, *t* = 2.274, effect size 0.608) lower in SV condition than N condition by 0.8 points on the 6‐20 Borg scale. Generalized linear mixed model analysis revealed significant interaction effects in sum hand motor function, bradykinesia and tremor sum, and resting and postural tremor items (*p* < 0.05), driven by improvements in symptoms post‐SV exercise. No significant interactions were observed for hand amplitude, rhythm of movement, or lower body symptoms. Overall, data support SV treadmill training as an exercise paradigm for PwPD to acutely impact hand motor function utilizing a standard treadmill. Future work should evaluate multiple sessions of training and evaluate dose–response effect of speed variability.

## 1. Introduction

Parkinson’s disease (PD) is the second‐most common neurodegenerative disease, with global age‐standardized incidence more than doubling between 1990 and 2021 [1, 2]. Hallmark motor signs include tremor, rigidity, and slowness of movement caused by reductions in central dopamine production [3]. Diagnosis is based primarily on motor function, with UK Brain Bank criteria evaluating bradykinesia, resting tremor, rigidity, and postural instability [4]. Motor symptoms typically appear on one side of the body and progress to bilateral involvement over time. The Unified Parkinson’s Disease Rating Scale Part III, or UPDRS‐III, is the ‘gold standard” for screening for PD‐specific motor function and determining symptom severity [5]. Tasks including repetitive hand and finger movements, tremor assessments, and walking are scored on a 0–4 point scale, with higher scores representing more severe symptoms. Assessment of motor function may also be conducted with validated wearable technology.

It has been well documented that exercise, specifically aerobic exercise, is disease modifying, may slow progression of PD chronically, and improve motor function acutely [6–8]. Current Parkinson’s Foundation and American College of Sports Medicine guidelines are clear in regard to exercise intensity, duration, and frequency: ACSM recommends at least 3 days per week of 30 min of moderate‐to‐vigorous aerobic exercise, 2‐3 days per week of resistance training, and 2‐3 days per week of cognitively challenging or balance/agility training [9]. Specifically, several clinical trials have supported the efficacy of moderate‐to‐vigorous aerobic activity in slowing disease progression and improving motor function. The recent SPARX2 clinical trial demonstrated that exercising for 6 months and 4 days weekly at a vigorous intensity resulted in a significant and clinically relevant 2.9 point difference in UPDRS‐III score compared to nonexercisers [10]. Likewise, the Park‐in‐Shape trial resulted in a 2.9 point UPDRS‐III score difference between exercisers and nonexercisers, with training consisting of stationary bicycling three times weekly at a moderate‐to‐vigorous intensity for 6 months [11].

In addition to exercise frequency, duration, and intensity, the exercise type is the key to evaluate. Elements of sensory feedback further augment the beneficial effects of aerobic training in people with Parkinson’s disease (PwPD) [12–14]. Neuroplastic adaptations may be enhanced in exercise that includes feedback in the form of deliberate tasks or goals, with feedback providing a “bottom‐up” stimulus occurring simultaneously with the “top‐down” signals of continuous movement. Imaging supports this hypothesis, with increased activity in sensorimotor cortices during aerobic training with external feedback [15]. Sensory feedback may also be provided by the exercise equipment itself. A basic example occurs in the comparison of treadmill training versus overground walking [16]. Treadmill training, with the continual sensory feedback of belt movement, results in improved gait parameters and working memory following 5 weeks of exercise compared to speed‐ and intensity‐matched overground walking [17]. Indeed, a network meta‐analysis of 148 exercise randomized controlled trials (RCTs) in PwPD found that three months of treadmill training resulted in an average 8.16 point improvement in UPDRS‐III score, while all exercise interventions (41) combined resulted in a 3.32 point increase in UPDRS‐III score [18].

Augmenting treadmill training with additional proprioceptive stimuli, such as varying left and right belt speeds (split‐belt training) or perturbation (changing orientation of belt), increases sensory feedback and overground gait response to exercise in PwPD [19]. A 2022 review concluded that treadmill training with sensory feedback components resulted in greater improvements in gait than standard treadmill training in PwPD [19]. Examples include split‐belt treadmill training, with increased variance in left and right belt speeds linked to greater improvements [20, 21], and perturbation treadmill training, involving constant changes in orientation of surface while walking [22, 23]. Eight weeks of perturbation treadmill training significantly improved functional gait measures compared to standard treadmill training [22], with improvements in overground gait speed visible after a single session of perturbation treadmill training [24]. One bout of split‐belt treadmill training with variability in belt speed across left and right sides also positively impacts gait parameters [21], and 8 weeks of split‐belt treadmill training resulted in clinically meaningful improvements in the UPDRS‐III score [20]. Although aforementioned acute studies primarily evaluate overground gait response, the chronic response of UPDRS‐III scores to split‐belt treadmill training and dynamic cycling interventions (13.8% improvement in three sessions [25]) supports an acute investigation of the same symptoms. Collectively, these paradigms support the efficacy of proprioceptive feedback during aerobic exercise, whether chronic or acute.

However, existing sensory feedback exercise equipment is expensive and not typically available outside of a small number of medical or research facilities. With diagnosis rates increasing and the knowledge that exercise improves function, it is important to provide accessible and effective exercise programming for PwPD [3]. Thus, the purpose of this pilot study is to examine if a single session of enhanced sensory feedback during treadmill training using only the treadmill itself is (A) feasible in this population and (B) if the program drives acute changes in motor function. We hypothesize that KinesiaONE motor function scores, a subjective measure of UPDRS‐III symptoms, will improve following one bout of speed‐variable (SV), but not normal, unvaried speed (N), exercise.

## 2. Methods

### 2.1. Study Design

PwPD (*N* = 14, Table 1) completed 2 sessions of treadmill walking in a counterbalanced order. Odd‐numbered participants (*n* = 7) completed N exercise first, while even‐numbered participants (*n* = 7) completed SV exercise first. Participants were numbered in order of study clearance. Random sequence generation was not utilized for participant assignment. Both split‐belt and perturbation treadmill training involved an initial single‐session evaluation followed by later clinical trials, supporting the initial single‐session counterbalanced design of the current pilot study [20–24].

**TABLE 1:** Demographics.

	Mean	SD	Minimum	Maximum
Age (years)	67.6	6.5	57	75
BMI (kg/m^2^)	26.6	4.5	19.1	36
LEDD	520.6	255.3	0	988
PD Diagnosis (years)	3.4	3.2	0.8	13.7
Sex	8 male, 6 female			

*Note:* PD Diagnosis indicates time since formal diagnosis of idiopathic Parkinson’s disease.

Abbreviations: BMI = Body Mass Index, LEDD = Levodopa Equivalent Daily Dose, SD = standard deviation.

**FIGURE 1:**
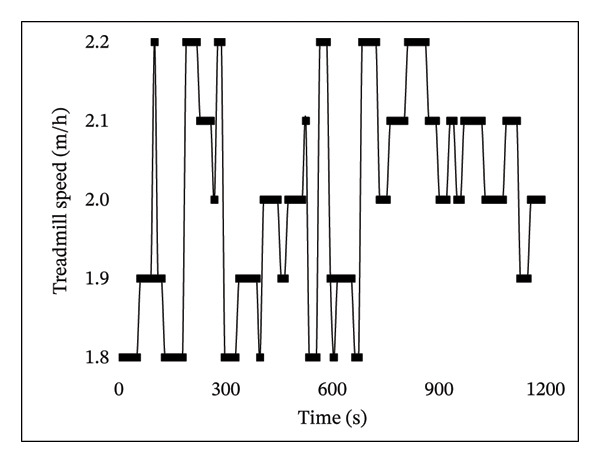
Speed variable treadmill training paradigm.

Each exercise session involved a 5‐min warmup, 20 min of exercise, and a 5‐min cooldown. One session (normal; N) involved consistent treadmill belt speed at a baseline speed, while the other session (SV) involved constant fluctuations in treadmill belt speed around baseline speed (Figure 1). SV condition included 24 speed changes every 10–60 s, with speeds varying −0.2, −0.1, 0.0, 0.1, or 0.2 mph from baseline with time‐series weighted average equaling baseline speed. The treadmill (Landice L10 Achieve, Landice, Inc., New Jersey, USA) was preprogrammed for both N and SV sessions, with segment change‐of‐speed beeps occurring at the exact time points per session to standardize audio feedback. Treadmill incline was kept at 0% for duration of exercise. A 5‐min warmup and 5‐min cooldown were conducted 0.5 mph below baseline speed at a consistent speed (e.g., no fluctuations during warmup/cooldown in SV condition). Fluctuations were chosen to minimize changes in cardiorespiratory exercise intensity between N and SV conditions, with timing of fluctuations (10–60 s) imitable in a gym or home setting. Baseline speed was set at 70% of participants’ overground walking speed during pre‐exercise 10 Meter Walk Test at normal speed mirroring perturbation treadmill walking [24, 26]. Baseline speeds ranged 1.8–2.8 miles per hour (mph), with median baseline speed of 2.05 mph.

Baseline speed is 2.0 mph. Speed fluctuates ≤ ± 0.2 mph from baseline every 10–60 s via automatic treadmill programming, with the time‐series average of all speeds equal to baseline.

### 2.2. Participant Characteristics

Participants were ON medication and instructed to keep activity levels, medication timing, and footwear consistent across visits. Visits were conducted at the same time of 3–14 days apart (72+ hours), with washout period selected to minimize potential carryover of initial session and control for potential chronic changes in cardiorespiratory fitness. A foundational work in the field utilized a 24‐h carryover period between treadmill, treadmill simulation, and overground walking conditions, supporting the choice of washout period [16]. Inclusion criteria included idiopathic PD diagnosis, age 50–79 years, undiagnosed with neurological conditions other than PD, and confidence in ability to walk for 30 min on a treadmill without assistance. Participants with orthopedic injuries that affected exercise safety, deep brain stimulation (DBS), or 2+ falls in the month prior to screening were excluded. Participants were required to complete all assigned exercise for analysis. All participants met criteria for safe exercise using American College of Sports Medicine (ACSM) Exercise Preparticipation Screening. No participants regularly utilized walking aids (cane and wheelchair). Participants wore a fall belt and were required to hold handrails for duration of exercise.

### 2.3. Outcome Measures

Pre‐ and postexercise assessments included overground gait, cognitive function, and motor function tasks. KinesiaONE sensor (Great Lakes Neurotechnologies, Ohio, USA) provided an objective measure of motor function on participant reported most‐affected side. This device is validated for use with PwPD, with tasks and scoring modeling after the UPDRS‐III [27], and has been used in prior studies of variable exercise [28, 29]. A standardized verbal script was utilized for all instructions, with demonstrations and task time countdowns consistently visible on tablet screen. Each task was scored on a 0–4 scale, with higher scores indicating more severe symptoms. Tremor, bradykinesia, rhythm, and amplitude of hand movements were assessed across 7 tasks lasting 15 s each. A sum score (0–48 point scale) of all upper‐body tasks was taken, with subscores (0–12 point scales) of upper‐body categories (rhythm, amplitude, bradykinesia, and tremor) and individual scores (0–4 point scales) of tremor subtypes (resting, postural, and kinetic) tabulated. In addition, lower‐body tasks included toe taps, leg lifts, and PD‐specific gait. Participants completed 10 min of seated rest prior to exercise after completing all baseline assessments.

Participants wore a Polar H10 heart rate (HR) monitor for continuous assessment of HR and rating of perceived exertion (RPE) was evaluated using 6‐20 Borg scale every 5 min during exercise. Treadmill screen was covered for the duration of exercise. Participants were blinded to condition within reason (condition not revealed verbally, screen covered, and auditory stimuli matched). Following the completion of exercise, participants immediately repeated motor function assessments. One individual was excluded from interaction analysis due to missing data from N condition (equipment syncing malfunction). Data supporting study findings may be available from corresponding author upon reasonable request.

### 2.4. Analysis

SPSS Version 31.0 (IBM SPSS, New York, USA) was utilized to evaluate descriptive data (e.g. demographics) and changes in HR and RPE with paired samples *t*‐tests (alpha set at 0.05). Effect size is represented as Cohen’s d. Motor function data was evaluated with JASP (JASP Team, Amsterdam, Netherlands), a statistical platform based on R programming language. Generalized linear mixed models (GLMMs) were used to evaluate the effects of condition (SV vs. N), time (pre vs. post), and their interaction, with subject included as a random effect. Hand motor function sum score was specified a priori as the primary outcome. Subtype analyses were considered secondary and are reported without adjustment for multiple comparisons. Estimated marginal means (EMMs) allowed for comparison across condition and timepoint.

This study was approved by Kent State University’s Institutional Review Board (IRB #2285) on January 22, 2025. Full written informed consent was obtained from all participants.

## 3. Results

### 3.1. SV/N HR and Perceived Exertion

**FIGURE 2:**
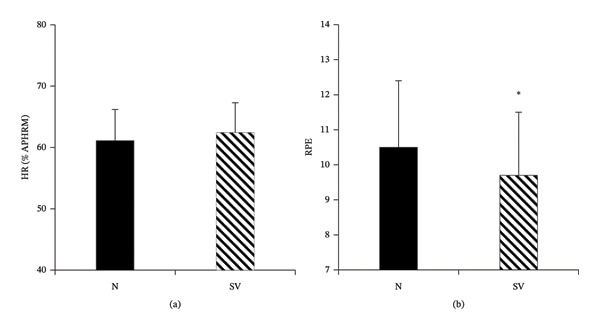
Average age‐predicted heart rate maximum percentage and rating of perceived exertion across SV and N sessions. (a) APHRM = age‐predicted heart rate maximum (220‐age). One participant excluded due to equipment malfunction. Error bars: standard deviation. (b) RPE = Borg rating of perceived exertion (6‐20 scale). ^∗^Significant (*p* < 0.05) difference. Error bars: standard deviation.

Paired samples *t*‐tests revealed no significant difference (*p* = 0.146, *t* = −1.102, *n* = 13, effect size: 0.306) in average HR (percentage of age‐predicted maximum; %APHRM; maximum estimated as 220‐age) across SV and N conditions (Figure 2a). Participants rated SV condition to require significantly (*p* = 0.020, *t* = 2.274, *n* = 14) less effort by 0.8 points or 7.6%, with a medium‐to‐large effect size of 0.608 (Figure 2b). Both SV and N exercise efforts are categorized by participants on average as “light” using Borg 6‐20 RPE scale reflecting average of four exercise timepoints. No adverse events including falls took place.

## 4. KinesiaONE Motor Function Outcomes

### 4.1. Hand Motor Function Sum

**FIGURE 3:**
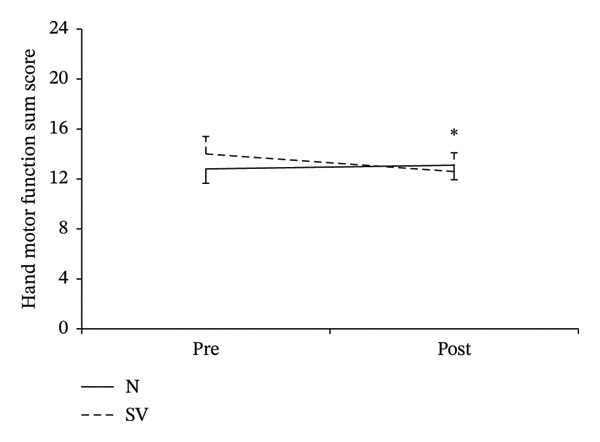
Hand motor function response to SV and N exercise. Error bars: standard error of mean. ^∗^Denotes significant interaction effect (*p* = 0.007).

KinesiaONE hand movement sum score (0–48 scale), representing the sum of assessed hand function categories (tremor, bradykinesia, amplitude, and rhythm) demonstrated a significant interaction effect (*p* = 0.007, *X* ^2^ = 7.274, SE = 0.219, *n* = 12, Figure 3), with EMM pre–post exercise comparison revealing SV condition average symptom improvement of 9.7% and N condition average symptom worsening of 8.2% postexercise. Ten out of 13 individuals experienced improvements in sum score following SV exercise. Twelve individuals had SV and N comparisons mapped, while one individual had missing item data from N condition and was able to be evaluated in SV condition solely.

### 4.2. Hand Motor Function Subtypes: Bradykinesia, Tremor, Rhythm, and Amplitude

**FIGURE 4:**
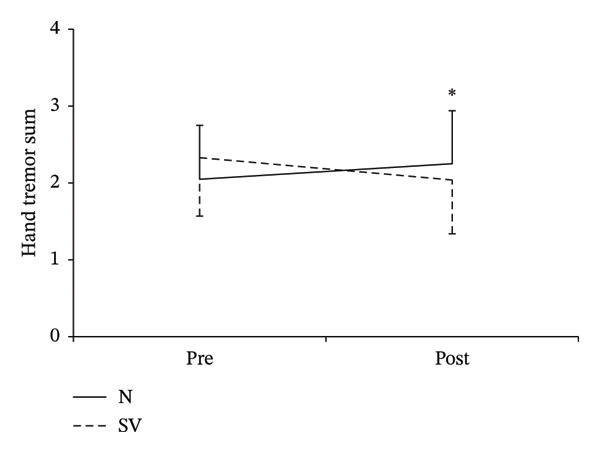
Hand tremor response to SV and N exercise. ^∗^Significant (*p* = 0.019) interaction effect. Error bars: standard error of mean.

Hand tremor sum score (postural, kinetic, and resting tremors; 0–12 scale) demonstrated a significant interaction effect (*p* = 0.019, *X* ^2^ = 5.548, SE = 0.126, *n* = 13; Figure 4). Ten out of 13 participants experienced improvement in tremor sum score following SV exercise. EMM revealed average improvement by 12.4% following SV exercise.

**FIGURE 5:**
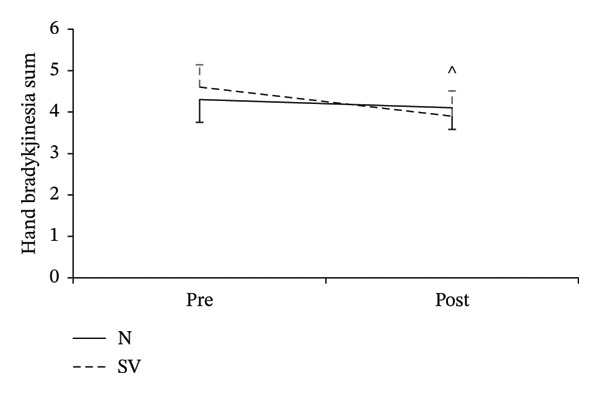
Bradykinesia response to SV and N exercise. ^^^ Significant (*p* = 0.049) main effect of time. Error bars: standard error of mean.

Bradykinesia (0–12 scale) subscore, tabulated by adding speed score from three distinct hand movements, had no significant interaction effect of program by time (*p* = 0.567, *X* ^2^ = 0.327, SE = 0.059, *n* = 13; Figure 5). However, a significant main effect of time was observed (*p* = 0.049, *X* ^2^ = 3.883, SE = 0.069, *n* = 13), with hand speed improving following both N and SV conditions by a pooled average of 6.6%.

No significant or notable interaction effects were observed for hand amplitude subscore (*p* = 0.194, *X* ^2^ = 1.689, SE = 0.077, *n* = 12) or hand rhythm subscore (*p* = 0.114, *X* ^2^ = 2.502, SE = 0.095, *n* = 12). One symptom, dyskinesia, showed a significant condition by time interaction (*p* = 0.003, *X* ^2^ = 8.527, SE = 0.015, *n* = 13), with dyskinesia worsening following SV exercise. However, levels were very low, with a post‐SV exercise average score of 0.24 out of 4 points and thus unlikely to greatly affect day‐to‐day function.

### 4.3. Hand Tremor Subtypes: Resting, Postural, and Kinetic

**FIGURE 6:**
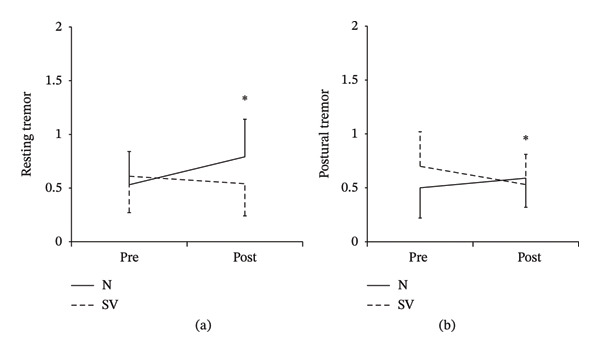
Resting and postural tremor response to SV and N exercise. (a) Resting hand tremor response to exercise. ^**∗**^Significant (*p* = 0.004) interaction effect. Error bars: standard error of mean. (b) Postural hand tremor response to exercise. ^∗^Significant (*p* = 0.019) interaction effect. Error bars: standard error of mean.

Resting tremor (0–4 scale) demonstrated a significant (*p* = 0.004, *X* ^2^ = 8.400, SE = 0.027, *n* = 13) interaction effect (Figure 6a). EMM revealed average improvement by 11.5% post‐SV exercise and average worsening by 49.1% post‐N exercise. Postural tremor (0–4 scale) showed a significant interaction effect (*p* = 0.019, *X* ^2^ = 5.485, SE = 0.028, *n* = 13, Figure 6b), with SV condition resulting in improvements by 24.1% and N condition resulting in worsening of tremor by 20.0%. There were no significant effects (*p* = 0.691, *X* ^2^ = 0.158, SE = 0.020, *n* = 13) observed for kinetic tremor.

#### 4.3.1. Lower‐Body Symptom Response

Lower‐body motor function was assessed with KinesiaONE sensor attached to heel of participant’s shoe. There were no significant interaction effects observed for toe tapping task (*p* = 0.093, *X* ^2^ = 2.819, SE = 0.022, *n* = 12), leg‐lifting task (*p* = 0.063, *X* ^2^ = 3.452, SE = 0.017, *n* = 12), or gait (*p* = 0.635, *X* ^2^ = 0.226, SE = 0.013, *n* = 12) assessing 5 m walking task at comfortable speed. One participant was excluded from lower‐body data analysis due to missing data from equipment malfunction at one timepoint.

## 5. Discussion

Average HR values support pilot study aim of evaluating sensory feedback across conditions without changing physiological exercise intensity between conditions. This finding is the key in laying the groundwork for future interventional analysis, confirming that exercise variability, not cardiorespiratory intensity, differentiated trials. The lack of significant or clinically meaningful difference in HR across conditions reinforces that changes of  ≤ 0.2 mph from baseline speed do not impact exercise cardiorespiratory intensity or increase cardiovascular strain. Furthermore, participants’ average exercise intensity fell in the 60%–65% APHRM range, indicating moderate‐intensity aerobic training. Additionally, lower RPE scores in SV condition support the potential for SV exercise to lower a barrier to exercise engagement by requiring significantly less perceived effort despite matched physiological response.

Collectively, significant and near‐significant groups by time interactions signal efficacy of SV exercise in impacting acute PD motor function. Author’s hypothesis that motor function would improve following SV but not following N exercise was partially supported in upper‐body PD function scores although not supported in lower‐body PD function scores. Sum KinesiaONE hand score, indicating severity of tremor, bradykinesia, rhythm disturbance, and amplitude across multiple hand movements, demonstrated a significant difference across conditions with benefit concentrated in the SV condition. Notably, effects existing for hand motor function support a nonlocal effect of SV treadmill exercise, as participant’s upper body was kept still on railings for a duration of exercise per instructions while the musculature of lower body was active.

Further subscores and individual KinesiaONE item scores reveal specifically that hand tremor responded acutely and beneficially to one bout of SV exercise but not N exercise, while hand speed improved following both SV and N conditions. Hand movement amplitude, hand rhythm, and PD‐specific gait measures did not demonstrate interaction or main effects across SV or N conditions. When evaluating further contributions to symptom change, differences in tremor response indicated potential neural response differing by tremor subtype. Resting and postural tremor demonstrated significant interaction with improvements in SV condition while kinetic tremor did not change significantly or meaningfully across conditions or timepoints. Although prior literature demonstrated acute benefits of treadmill exercise on gait, observed declines in motor function following N condition may be due to a wearing‐off effect of levodopa over exercise session. It is important to note that some group interactions may be, in fact, due to worsening in function following N condition, not improvement following SV condition. In summary, hand motor function, driven by improvements in resting tremor and postural tremor, responded acutely and positively to a bout of SV exercise.

This specific pattern of hand symptom and tremor response has been observed in similar work involving dynamic cycling in people with PD. Hand tremor and movement speed significantly improved following one session of assisted cycling at a high cadence [30], while hand amplitude and rhythm did not respond to the single session of assisted cycling, a pattern very similar to the current study’s findings. Resting tremor and bradykinesia can be classified as dopamine‐sensitive symptoms, providing insight into potential mechanism of improvement following sensorimotor aerobic exercise [29]. The mirrored symptom response pattern in dynamic cycling, a well‐supported intervention [28, 29, 31, 32], supports this pilot study’s aim of elucidating sensorimotor feedback with a standard treadmill via SV training.

The next step in analysis of SV training is to evaluate multiple sessions: in a clinical trial of assisted cycling over 6 sessions, UPDRS‐III motor function significantly (*p* = 0.002) improving by 17% [28]. It is notable that average HR was in 60%–65% APHRM range in aforementioned cycling trial, mirroring the current study’s exercise intensity. Furthermore, dopamine‐sensitive PD symptoms improved significantly (*p* < 0.001) following an even longer trial (12 sessions) of assisted cycling exercise with resistance progression, supporting potential targeting of tremor and bradykinesia with proprioceptive aerobic exercise [29]. Collectively, links between acute and chronic responses in similar exercise paradigms support future evaluation of SV treadmill training as an exercise intervention. Unfortunately, primary outcomes of treadmill exercise paradigms involving sensorimotor interaction (perturbation, split‐belt, and standard treadmill) are concentrated in overground gait measures, thus not allowing for direct comparison of acute motor function changes with current findings [21, 24, 26].

Limitations of the current study include a relatively small sample size (*n* = 14) and a highly active population. Ten of the 14 participants reported high physical activity levels on the International Physical Activity Questionnaire (IPAQ, [33]), with only 1 individual reporting low physical activity. Although no adverse events (e.g., falls) took place throughout the study, two cases provide guidance for future work and application. An individual reporting low physical activity was excluded from present analysis due to inability to complete exercise due to aerobic intensity, potentially indicating that this paradigm is best suited to individuals with at least moderate activity levels. Furthermore, one individual cleared all screening but was unable to complete the warmup period on treadmill (five minutes without speed changes 0.5 mph below baseline speed) due to concerns over maintenance of balance, indicating that an additional screening question assessing balance and/or prior treadmill experience may be necessary. Additionally, treadmill exercise is likely unsuitable for individuals with higher fall risk, inability to walk independently, and/or more advanced PD, potentially limiting usage. Statistically, secondary outcomes of lower‐body motor function were evaluated without adjustment. Allocation of participants was quasirandom by order of consent.

Overall, this work supports future evaluation of multiple sessions of SV treadmill training. Evaluation of dopamine‐sensitive vs. non‐dopamine‐sensitive response will allow for individual targeting of exercise prescription if current patterns of response (resting tremor, postural tremor and movement speed) hold. Additionally, dose–response effect of speed variability may be assessed via increasing time‐series frequency of speed changes to best determine exercise prescription. However, in this paradigm, it is key to keep the frequency of speed changes realistic for application by individuals in a gym or home setting, e.g., fluctuations not exceeding every ∼10 s. In summary, active PwPD, if able to confidently and safely walk on a treadmill, may be encouraged to vary belt speed frequently and at a low amplitude to maximize acute resting tremor, postural tremor, and movement speed response from treadmill walking.

## Funding

This project was supported by Kent State University’s Research Award.

## Disclosure

A preliminary version of this work appeared as a graduate thesis, available through ProQuest https://www.proquest.com/docview/3283136878 [34]. The present paper has been revised and adapted for publication.

## Conflicts of Interest

The authors declare no conflicts of interest.

## Data Availability

The data that support the findings of this study are available from the corresponding author upon reasonable request.
